# Antibiofilm Activities of Cinnamaldehyde Analogs against Uropathogenic *Escherichia coli* and *Staphylococcus aureus*

**DOI:** 10.3390/ijms23137225

**Published:** 2022-06-29

**Authors:** Yeseul Kim, Sanghun Kim, Kiu-Hyung Cho, Jin-Hyung Lee, Jintae Lee

**Affiliations:** 1 School of Chemical Engineering, Yeungnam University, Gyeongsan 38541, Korea; yeseul@ynu.ac.kr (Y.K.); minimo017@ynu.ac.kr (S.K.); 2 Gyeongbuk Institute for Bioindustry, Andong 36618, Korea; khcho68@gmail.com

**Keywords:** antibiofilm, cinnamaldehyde, *Staphylococcus aureus*, uropathogenic *Escherichia coli*

## Abstract

Bacterial biofilm formation is a major cause of drug resistance and bacterial persistence; thus, controlling pathogenic biofilms is an important component of strategies targeting infectious bacterial diseases. Cinnamaldehyde (CNMA) has broad-spectrum antimicrobial and antibiofilm activities. In this study, we investigated the antibiofilm effects of ten CNMA derivatives and *trans*-CNMA against Gram-negative uropathogenic *Escherichia coli* (UPEC) and Gram-positive *Staphylococcus aureus*. Among the CNMA analogs tested, 4-nitrocinnamaldehyde (4-nitroCNMA) showed antibacterial and antibiofilm activities against UPEC and *S. aureus* with minimum inhibitory concentrations (MICs) for cell growth of 100 µg/mL, which were much more active than those of *trans*-CNMA. 4-NitroCNMA inhibited UPEC swimming motility, and both *trans*-CNMA and 4-nitroCNMA reduced extracellular polymeric substance production by UPEC. Furthermore, 4-nitroCNMA inhibited the formation of mixed UPEC/*S. aureus* biofilms. Collectively, our observations indicate that *trans*-CNMA and 4-nitroCNMA potently inhibit biofilm formation by UPEC and *S. aureus*. We suggest efforts be made to determine the therapeutic scope of CNMA analogs, as our results suggest CNMA derivatives have potential therapeutic use for biofilm-associated diseases.

## 1. Introduction

Bacterial biofilms are surface-attached communities of bacteria encased in self-generated extracellular polymeric substances. Microbial biofilms are widespread in nature, and are closely related to infectious diseases. Biofilms allow bacteria to survive in hostile environments [1], and are often resistant to antibiotics and host defenses; thus, they contribute to chronic infections [2]. In particular, the prevention of pathogenic biofilm formation on food and surfaces, including those of medical devices, are of considerable importance. Furthermore, since it was reported that aminoglycoside antibiotics often promote biofilm formation [3], identifying novel antibiofilm compounds has become a priority [4]. Recently, many compounds endowed with potent in vitro antibiofilm activity have been described, but, unfortunately, no derivative is close to clinical trial [5,6].

*Trans*-cinnamaldehyde (*trans*-CNMA) is a naturally occurring antimicrobial and antibiofilm agent that exhibits activities against commensal, enterohemorrhagic, and uropathogenic *Escherichia coli* (UPEC) strains [7,8,9] and methicillin-resistant *Staphylococcus aureus* strains [10,11]. Recently, the antibiofilm effects of several CNMA analogs on *Streptococcus pyogenes* [12], *Vibrio* species [13], and *Candida albicans* strains [14] have been reported.

Gram-negative UPEC is a major pathogen of the urinary tract that expresses structural (fimbriae, pili, and curli) and secreted toxins (iron-acquisition systems), and virulence factors [15]. Gram-positive *S. aureus* is the primary agent of acute and chronic infections; drug-resistant *S. aureus* has become a serious problem [16]. Furthermore, biofilm formation by UPEC or *S. aureus* crucially facilitates the persistence of chronic infections due to the inherent tolerance of biofilms to common antibiotics. Hence, we investigated the antibiofilm and antimicrobial effects of *trans*-CNMA and ten CNMA derivatives against UPEC and *S. aureus*. To understand the mechanisms responsible for their effects, morphologies were investigated by scanning electron microscopy (SEM) and virulence factor assays (motility, cell surface hydrophobicity, hemolysis, and staphyloxanthin assays). In addition, the antibiofilm efficacies of selected CNMA derivatives were investigated in a mixed UPEC/*S. aureus* biofilm model.

## 2. Results

### 2.1. Antibiofilm and Antimicrobial Activities of Trans-CNMA and Its Derivatives against UPEC and S. aureus

**Figure 1:**
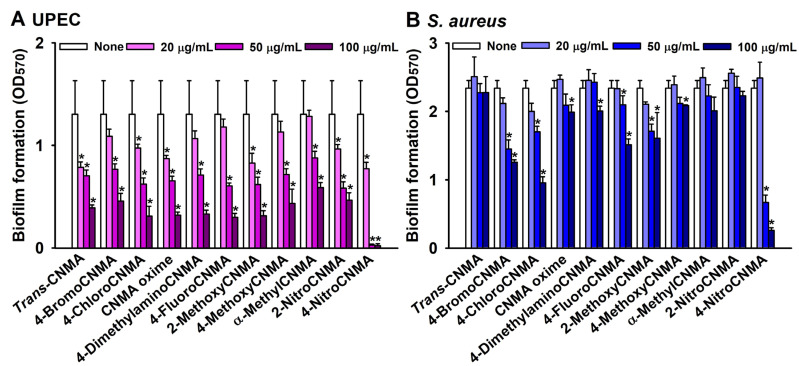
Antibiofilm activities of *trans*-cinnamaldehyde (*trans*-CNMA) and its derivatives. Antibiofilm screening against UPEC (**A**) and *S. aureus* (**B**). Error bars indicate standard deviations. * *p* < 0.05 vs. nontreated controls (None).

*Trans*-CNMA and ten CNMA derivatives were initially screened for antibiofilm activity against UPEC and *S. aureus* at concentrations of 20, 50, and 100 µg/mL. UPEC biofilm formation was more sensitive to CNMAs than *S. aureus* biofilm formation (Figure 1A,B). The majority of CNMAs at 50 or 100 µg/mL significantly inhibited UPEC biofilm formation, though 4-nitroCNMA was the most active at 20 and 50 µg/mL (Figure 1A). For example, the backbone molecule *trans*-CNMA at 50 µg/mL inhibited UPEC biofilm formation by 46%, whereas 4-nitroCNMA at 50 µg/mL inhibited biofilm formation by 98%. Regarding *S. aureus* biofilm formation, 4-bromoCNMA, 4-chloroCNMA, and 4-nitroCNMA at 100 µg/mL inhibited biofilm formation by more than 46%, whereas *trans*-CNMA at the same concentration had no inhibitory effect (Figure 1B).

**Figure 2:**
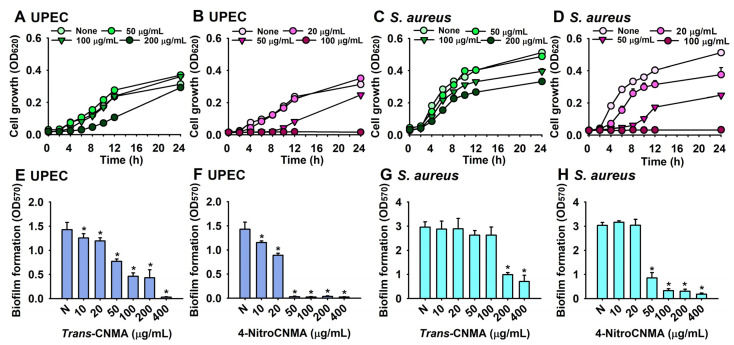
Planktonic cell growth in the presence of *trans*-CNMA or 4-nitroCNMA against UPEC in NB medium (**A**,**B**) and *S. aureus* in LB medium (**C**,**D**). Antibiofilm activities of *trans*-CNMA and 4-nitroCNMA against UPEC (**E**,**F**) and *S. aureus* (**G**,**H**). Error bars indicate standard deviations. * *p* < 0.05 vs. nontreated controls (None).

The minimal inhibitory concentrations (MICs) of the eleven CNMAs against the planktonic cell growths of UPEC and *S. aureus* were determined under static conditions (Table 1). The MICs of most CNMAs were >400 µg/mL, but 4-nitroCNMA had an MIC of 100 µg/mL against both UPEC and *S. aureus*, and 4-chloroCNMA and 4-fluoroCNMA had MICs of 200 µg/mL against UPEC. Furthermore, the growth curves of planktonic cells confirmed that 4-nitroCNMA at 100 µg/mL significantly prevented planktonic cell growth by UPEC and *S. aureus*, whereas *trans*-CNMA at 200 µg/mL slightly retarded bacterial growth (Figure 2A–D). Due to its potent antimicrobial and antibiofilm activities, 4-nitroCNMA was selected for further assays, and *trans*-CNMA was used as a structural control.

The results of a more detailed biofilm study showed that *trans*-CNMA and 4-nitroCNMA dose-dependently inhibited biofilm formation by UPEC and *S. aureus* (Figure 2E–H). For example, *trans*-CNMA at 100 µg/mL inhibited UPEC biofilm formation by 70%, while 4-nitroCNMA at 50 µg/mL (50% of its MIC) inhibited biofilm formation by more than 98% (Figure 2E,F). *Trans*-CNMA at 100 µg/mL did not inhibit *S. aureus* biofilm formation, whereas 4-nitroCNMA at 100 µg/mL (1 × MIC) inhibited it by 89% (Figure 2G,H).

The biofilm dispersing activities of *trans*-CNMA and 4-nitroCNMA against preformed UPEC and *S. aureus* biofilms were investigated. Up to 400 µg/mL (a concentration four to eight times higher than that required for biofilm inhibition), 4-nitroCNMA did not disrupt established biofilms of UPEC or *S. aureus* (Supplementary Figure S1). This result confirms that biofilm dispersal is more difficult than biofilm inhibition.

### 2.2. Microscopic Observations of Biofilm Formation by UPEC and S. aureus

**Figure 3:**
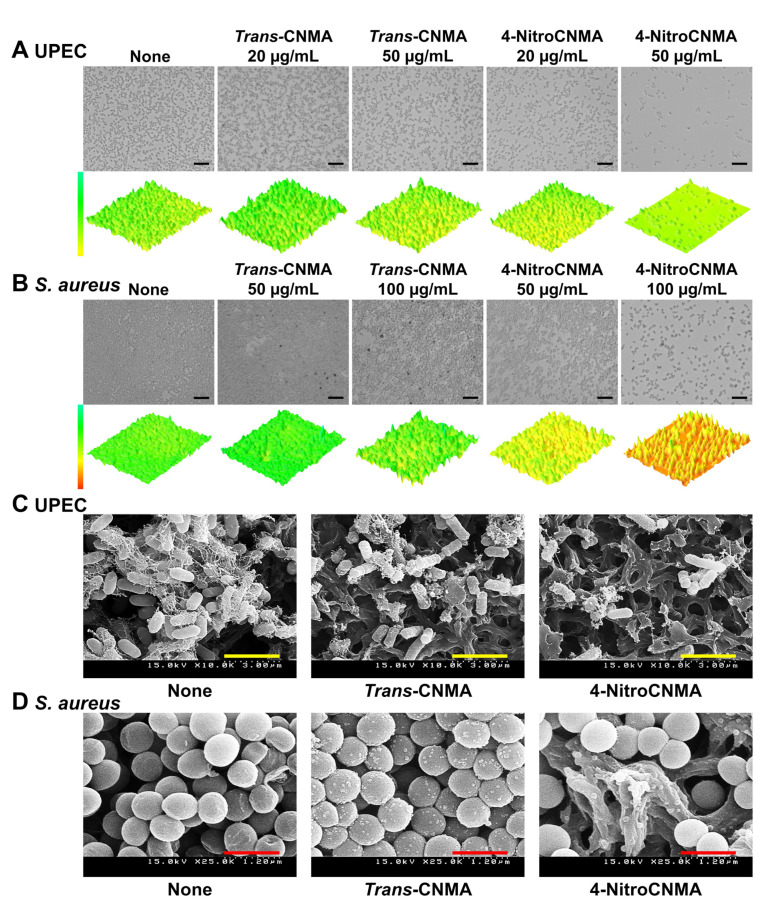
Microscopic observations of biofilm inhibition by *trans*-CNMA and 4-nitroCNMA. The 3D optical microscopy images of biofilm formation by UPEC (**A**) or *S. aureus* (**B**) in the presence or absence of *trans*-CNMA or 4-nitroCNMA. Black scale bars indicate 10 µm. SEM images of UPEC (**C**) and *S. aureus* (**D**) biofilms formed in the presence or absence of *trans*-CNMA and 4-nitroCNMA at 50 µg/mL. Yellow and red scale bars indicate 3 and 1.2 µm, respectively.

Microscopic observations confirmed the antibiofilm efficacies of *trans*-CNMA and 4-nitroCNMA (Figure 3A,B). Crystal violet biofilm assay results (Figure 2E–H) showed *S. aureus* formed thicker biofilms than UPEC in 96-well polystyrene plates. *Trans*-CNMA and 4-nitroCNMA both dose-dependently inhibited biofilm formation by UPEC (Figure 3A) and *S. aureus* (Figure 3B), though 4-nitroCNMA was considerably more potent than *trans*-CNMA.

SEM was used to observe biofilm cells of UPEC and *S. aureus* on nylon membrane pieces. *Trans*-CNMA and 4-nitroCNMA at 50 µg/mL reduced the number of UPEC biofilm cells (Figure 3C). *Trans*-CNMA and 4-nitroCNMA notably reduced fimbriae production compared with untreated controls, which produced entangled fimbriae that aggregated UPEC cells. The treatment of 4-nitroCNMA diminished the number of *S. aureus* biofilm cells (Figure 3D).

### 2.3. Trans-CNMA and 4-nitroCNMA Affected the Swimming Motility of UPEC

**Figure 4:**
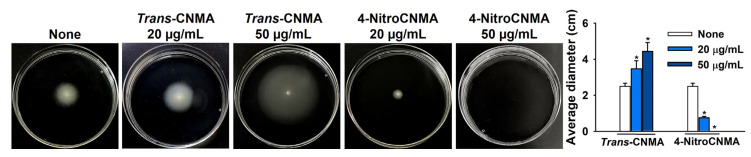
Swimming motility of UPEC in the presence of *trans*-CNMA or 4-nitroCNMA. Mean swimming motility was quantified using halo diameters. * *p* < 0.05 vs. nontreated controls (None).

Motility plays an important role in *E. coli* biofilm formation [17,18]; thus, we examined the effects of *trans*-CNMA and 4-nitroCNMA on the swimming motility of UPEC. Interestingly, *trans*-CNMA at 50 µg/mL significantly increased the swimming motility of UPEC, whereas, at the same concentration, 4-nitroCNMA abolished swimming motility (Figure 4). The results suggest that the antibiofilm activity of 4-nitroCNMA is partly due to its inhibitory effect on swimming motility.

### 2.4. Effects of Trans-CNMA and 4-nitroCNMA on Cell Hydrophobicity and Hemolysis

Cell hydrophobicity plays a role in cell adhesion because hydrophobic cells better adhere to hydrophobic surfaces [19]. However, *trans*-CNMA and 4-nitroCNMA at concentrations up to 50 µg/mL did not change the hydrophobicities of UPEC or *S. aureus* (Supplementary Figure S2).

**Figure 5:**
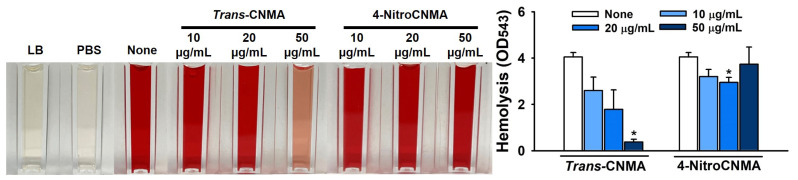
Inhibition of *S. aureus* induced hemolysis by *trans*-CNMA and 4-nitroCNMA. Sheep red blood cell hemolysis by *S. aureus* was investigated in the presence or absence of *trans*-CNMA or 4-nitroCNMA after culture for 24 h. The images show hemolysis activities in spectrophotometer cuvettes. * *p* < 0.05 vs. nontreated control (None).

*S. aureus* produces *α*-toxin, which hemolyzes sheep red blood cells [20,21]; this activity is positively correlated with biofilm formation [22]. Interestingly, *trans*-CNMA at 50 µg/mL significantly reduced hemolysis by *S. aureus*, whereas 4-nitroCNMA at concentrations of 20 µg/mL had a slight inhibitory effect (Figure 5). This result supports the previous observations that *trans*-CNMA inhibits *S. aureus* adherence to latex and its ability to lyse erythrocytes [23].

Additionally, we investigated the effects of *trans*-CNMA and 4-nitroCNMA on the production of staphyloxanthin (a yellow pigment), an important immune evasive virulence factor of *S. aureus*. However, *trans*-CNMA and 4-nitroCNMA at concentrations up to 50 µg/mL did not affect staphyloxanthin production in *S. aureus* (Supplementary Figure S3).

### 2.5. Antibiofilm Activities of CNMAs against Mixed UPEC and S. aureus Biofilms

Various microbes coexist and form multispecies biofilms, further increasing tolerance to antimicrobial agents [24,25]. Initially, we developed a dual biofilm model of UPEC and *S. aureus* because it has been reported that *S. aureus* biofilm cells dominated other species such as UPEC, *Pseudomonas aeruginosa,* and *C. albicans* in previous multispecies biofilm models [26,27,28].

**Figure 6:**
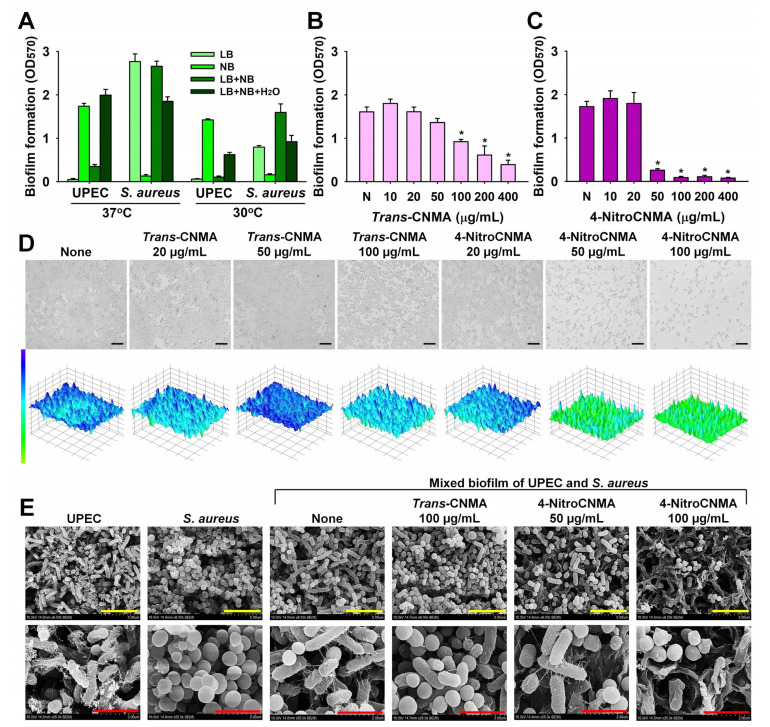
Antibiofilm activities of *trans*-CNMA and 4-nitroCNMA against mixed UPEC/*S. aureus* biofilms. Optimization of culture medium at 37 and 30 °C (**A**). Antibiofilm effects of *trans*-CNMA (**B**) and 4-nitroCNMA (**C**) on UPEC/*S. aureus* biofilms for 24 h. * *p* < 0.05 vs. nontreated controls (None). The 2D and 3D images of UPEC/*S. aureus* biofilms (**D**) after culturing in the presence of *trans*-CNMA or 4-nitroCNMA for 24 h. The black scale bar represents 10 µm. SEM images (**E**) of UPEC/*S. aureus* biofilms formed in the presence or absence of *trans*-CNMA or 4-nitroCNMA. Yellow and red scale bars indicate 5 and 2 µm, respectively.

Media composition was optimized first (Figure 6). Several researchers have reported that UPEC and *S. aureus* grow and form biofilms in NB and LB media, respectively, which we observed [26,27,28]. However, we found UPEC could not form biofilms in LB medium and that *S. aureus* could not grow or form biofilm in NB medium. In a 1:1 NB/LB medium, *S. aureus* formed biofilms, but UPEC did not (Figure 6A), which supports the findings in previous reports [26,27,28]. Furthermore, similar results were obtained at 37 °C and 30 °C (Figure 6A). Interestingly, when the 1:1 medium was diluted with water (to 1:1:1 NB:LB:water), UPEC and *S. aureus* formed strong biofilms at both 37 °C and 30 °C (Figure 6A); thus, this medium was used as the mixed UPEC/*S. aureus* biofilm model.

Under the optimized conditions, *trans*-CNMA and 4-nitroCNMA were found to dose-dependently inhibit mixed biofilm formation by UPEC and *S. aureus* (Figure 6B,C). For example, *trans*-CNMA at 100 µg/mL inhibited dual biofilm formation by 43%. In comparison, 4-nitroCNMA at 50 µg/mL (1/2 × MIC) inhibited it by 85%, which was similar to that observed in single-biofilm models (Figure 2). Microscopic observations confirmed *trans*-CNMA and 4-nitroCNMA inhibited mixed biofilm formation (Figure 6D), although 4-nitroCNMA was much more potent. The results of SEM analysis showed that UPEC and *S. aureus* were equally present in nontreated biofilms (Figure 6E). Notably, 4-nitroCNMA at 50 µg/mL markedly reduced UPEC and *S. aureus* attachments, inhibited UPEC fimbriae production, damaged UPEC cell membranes, and diminished numbers of *S. aureus* biofilm cells (Figure 6E).

## 3. Discussion

*Trans*-CNMA is produced by trees of the genus *Cinnamomum*, and is generally recognized as safe (GRAS) [29]; it is commonly used as a flavoring agent and in medical products, cosmetics, and perfumes [30,31]. *Trans*-CNMA has been well-reported to possess antimold, neuroprotective, antioxidant, anticancer, cardioprotective, anti-inflammatory, antifungal, and antibacterial properties [30,31]. Recently, the antibiofilm activity of *trans*-CNMA has been widely reported across an array of Gram-positive and -negative bacteria and fungal species [12,13,14]. In this study, we found the antibiofilm and antimicrobial activities of *trans*-CNMA and 4-nitroCNMA on Gram-negative uropathogenic *E. coli* (UPEC), Gram-positive *S. aureus*, and UPEC/*S. aureus* mixed biofilms.

The mechanisms responsible for the antimicrobial and antibiofilm activities of *trans*-CNMA and its derivatives are microbe-type-dependent [30,31]. It was reported that *trans*-CNMA at a high concentration (0.31 mg/mL) caused membrane lysis in *E. coli* and *S. aureus* strains [32]. At the molecular level, *trans*-CNMA reduces the expressions of the *fimA*, *fimH*, *focA*, *sfaA*, *sfaS*, and *papG* genes, which are involved in UPEC attachment and the invasion of host tissue [33]; downregulates curli genes; inhibits biofilm formation by enterohemorrhagic *E. coli* [8,9]; and reduces the adhesion of enteroaggregative *E. coli* on HEp-2 cells [34]. Furthermore, the present results support previously suggested mechanisms of *trans*-CNMA and 4-nitroCNMA inhibiting UPEC motility, fimbriae production, and biofilm formation (Figure 3 and Figure 4).

*Trans*-CNMA at sub-MIC levels has been reported to inhibit *S. aureus* biofilm formation partly by suppressing transcriptional regulator (*sarA)* [35]; repressing laminin-binding protein (*eno*), elastin-binding protein (*ebps*), and fibrinogen-binding protein (*fib*) [36]; and inhibiting the hemolytic activity of *S. aureus* and reduce its adherence [23]. In addition, *trans*-CNMA synergistically augments the effects of antibiotics on *S. aureus* [37]. However, the mechanism responsible for the *S. aureus* biofilm inhibition by *trans*-CNMA has not been clearly elucidated. Although we confirmed that *trans*-CNMA inhibited *S. aureus* induced hemolysis, the more active antibiofilm compound 4-nitroCNMA did not affect the hemolytic activity of *S. aureus* (Figure 5).

In this study, three halogenated CNMAs, two methoxyCNMAs, two nitroCNMAs and other CNMA derivatives were investigated (Table 1). Among these, three CNMA derivatives (4-bromoCNMA, 4-chloroCNMA, and 4-nitroCNMA) inhibited biofilm formation by UPEC and *S. aureus* (Figure 1). 4-NitroCNMA was most active against biofilm formation by UPEC and *S. aureus*, and 4-bromoCNMA and 4-chloroCNMA were active, but to lesser extents. 4-NitroCNMA had the lowest MIC (100 µg/mL) against UPEC and *S. aureus* (Table 1). Notably, 4-nitroCNMA at a sub-MIC concentration (50 µg/mL) inhibited biofilm formation by UPEC and *S. aureus* by more than 98% and 71%, respectively (Figure 2). Molecular studies are required to determine the mechanism involved. Recently, 4-nitroCNMA, 4-chloroCNMA, and 4-fluoroCNMA were reported to inhibit biofilm formation by Gram-negative *Vibrio parahaemolyticus* by repressing the expressions of quorum sensing and biofilm-related genes (*aphA*, *cpsA*, *luxS*, and *opaR*) [13]; *α*-methylCNMA and *trans*-4-methylCNMA inhibited biofilm formation by fungal *C. albicans* by downregulating hyphae related genes (*ECE1*, *IFD6*, *RBT5*, *UCF1*, and *UME6*) [14]. Based on our results, it appears that the antibiofilm activity patterns of CNMA derivatives and *trans*-CNMA are similar to those observed for CNMA derivatives against *V. parahaemolyticus* [13] (Figure 1) but differ from those observed against *C. albicans* [14]. Although speculative, the electron-withdrawing characteristics and positions of substituents on *trans*-CNMA may affect antimicrobial and antibiofilm activities, as substitution of electron withdrawing halogens (Br and Cl) and nitrogen on the four position of *trans*-CNMA were found to increase the electrophilicity, which enhanced antibacterial activity [38]. Similarly, 4-bromoCNMA, 4-chloroCNMA, and 4-nitroCNMA exhibited high antimicrobial and antibiofilm activities, whereas 2-nitroCNMA did not (Figure 1 and Table 1).

Despite the promising antimicrobial and antibiofilm activities of *trans*-CNMA and its derivatives against various microbes, their low water solubilities limit their applications [30,31]. To address this solubility issue, modified delivery systems, such as CNMA nanoemulsions [39], chitosan-CNMA dynagels [40], gold nanocarriers [41], CNMA liposomes [42], and polymeric systems loaded with CNMA, can be utilized. The use of *trans*-CNMA as an antibiofilm agent is limited by its cytotoxicity [43]. One study reported that CNMA derivatives exhibit nematicidal effects, but 4-nitroCNMA was found to have drug-like properties and did not violate the ‘rule-of-five’ [14]. Hence, we suggest further in vivo studies be conducted on the cytotoxicity and safety of CNMAs.

The antimicrobial and antibiofilm activities of *trans*-CNMA and its analogs have been widely reported. Our findings demonstrate that 4-nitroCNMA is much more potent than *trans*-CNMA at inhibiting biofilm formation by UPEC and *S. aureus* individually or in combination. In addition, 4-nitroCNMA reduced fimbriae production and the swimming motility of UPEC, though the mechanism responsible in *S. aureus* was not elucidated. The current study shows that CNMA analogs represent an important resource for designing innovative drugs for treating of persistent bacterial infections.

## 4. Materials and Methods

### 4.1. Reagents and Culture Strains

*Trans*-CNMA, ten CNMA analogs (Table 1), dimethyl sulfoxide (DMSO), and crystal violet were purchased from Sigma-Aldrich (St. Louis, MO, USA), Combi Blocks, Inc. (San Diego, CA, USA), or TCI Co. (Tokyo, Japan). The uropathogenic *Escherichia coli* O6:H1 CFT073 (UPEC; ATCC 700928) strain and a methicillin-sensitive *S. aureus* strain (MSSA; ATCC 6538) were obtained from American Type Culture Collection (Manassas, VA, USA). Nutrient broth (NB) for UPEC and Luria-Bertani (LB) medium for *S. aureus* were used for all single-strain studies. All experiments were conducted at 37 °C except as indicated. DMSO was used for dissolving the eleven CNMAs and as the negative control, and at 0.1% (v/v), it did not inhibit bacterial growth or biofilm formation. Planktonic cell growths and turbidities were measured at 600 nm using an Optizen 2120 UV spectrophotometer (Mecasys Co., Ltd., Daejeon, Korea). MIC was determined as the lowest concentration that visually inhibited planktonic cell growth. All experiments were conducted in at least two independent cultures in triplicate.

### 4.2. Crystal Violet Biofilm Assay

Biofilm formation was assessed in 96-well microtiter plates (SPL Life Sciences, Pocheon, Korea) using crystal violet, as previously described [44]. Briefly, UPEC cells were inoculated into fresh NB broth (300 μL) with an initial turbidity of 0.05 at 600 nm (5 × 10^6^ CFU/mL), and *S. aureus* cells were inoculated into fresh LB broth (300 μL) with an initial turbidity of 0.05 at 600 nm (1.5 × 10^7^ CFU/mL). CNMAs were added at different concentrations (0, 10, 20, 50, 100, 200, or 400 µg/mL) and cultivated for 24 h at 37 °C under static conditions. To measure biofilm formation, biofilms were rinsed three times with distilled water, incubated with 0.1% (*g*/*v*) crystal violet for 20 min at room temperature, and solubilized in 95% ethanol after removing crystal violet and washing with distilled water three times. Absorbances were measured at 570 nm (OD_570_) using a Multiskan EX microplate reader (Thermo Fisher Scientific, Waltham, MA, USA). Biofilm formation results are the averages of two independent experiments performed using six replicate wells. For the biofilm dispersal assay, UPEC or *S. aureus* biofilms were formed as described above for 24 h. Preformed biofilms were rinsed with distilled water three times to remove nonadherent cells. Different concentrations of the CNMAs were added to each well of a 96-well plate containing fresh medium, incubated for 24 h at 37 °C, and stained with crystal violet, as described above. Results are presented as the means of at least two independent cultures.

### 4.3. Microscopic Observations of Biofilms

To observe biofilm formations by UPEC or S. aureus, biofilm cells were produced as mentioned above for 24 h at 37 °C. After incubation, planktonic cells were discarded by gentle washing with water three times, and biofilms were analyzed by live imaging microscopy using an iRiS™ Digital Cell Imaging System (Logos BioSystems, Anyang, Korea). Biofilm images were regenerated as color-coded 2D and 3D images using ImageJ (https://imagej.nih.gov/ij/index.html accessed on 9 June 2022). UPEC and *S. aureus* biofilms on nylon filter membranes were examined by SEM, as previously described [45]. Briefly, a nylon filter membrane (Merck Millipore, Burlington, MA, USA) was cut into 0.5 × 0.5 cm pieces, autoclaved, and then a single piece was added to each well of 96-well plates having appropriate cell culture medium and incubated with or without CNMAs for 24 h at 37 °C. Biofilm cells on nylon membranes were washed with PBS, fixed with a mixture of glutaraldehyde (2.5%)/formaldehyde (2%) for 24 h, postfixed with OsO_4_ (osmium tetroxide), and dehydrated using ethanol and isoamyl acetate. After critical-point drying and sputter-coating, cells were imaged on membranes using an FE-SEM (field-emission scanning electron microscope) (S-4800; Hitachi, Tokyo, Japan) at 15 kV. All the experiments were conducted using at least two independent cultures.

### 4.4. Swimming Motilities of UPEC

Swimming motility was assessed using 0.23% agar plates containing 1% tryptone (*w*/*v*) and 0.25% NaCl (*w*/*v*) with or without CNMAs at 20 or 50 µg/mL. Fresh colonies of UPEC from LB agar plates were inoculated into 14 mL tubes containing 2 mL of LB medium and grown to an OD of 1.0 at 600 nm. Aliquots (0.2 µL) of these cultures were spotted on assay plates using sterilized micropipette tips, incubated for 24 h at 37 °C, and then average motility halo diameters were measured. All experiments were conducted using at least two independent cultures.

### 4.5. Cell Surface Hydrophobicities

Cell surface hydrophobicities were quantified as previously described [46]. Briefly, UPEC or S. aureus (1:100 dilution) were cultured overnight with or without CNMAs at 10, 20, or 50 µg/mL and then incubated with shaking at 250 rpm for 24 h at 37 °C. Cell suspensions (1 mL) were centrifuged at 7000× *g* for 5 min, and cells were disseminated in 1 mL of PBS. Hexadecane (300 µL) was then added to PBS buffered cell suspensions, vortexed vigorously for 90 s, and left undisturbed for 30 min at room temperature. PBS (1 mL, the blank) was processed in the same manner. OD values before vortexing (*A*_0_) and absorbances of aqueous phases (*A*_i_) were measured at 600 nm. Percent hydrophobicities were calculated using the following formula:

*Percent hydrophobicity* (%*H*) = (*A*_0_ − *A_i_*) × 100/*A_i_*

### 4.6. Staphyloxanthin Assay

The yellow color of staphyloxanthin enabled its production to be assessed by visual examination [47]. Briefly, *S. aureus* cells were inoculated at 1:100 dilution in LB (2 mL) and incubated for 24 h with or without CNMAs at 20 or 50 µg/mL at 37 °C in 14 mL tubes at 250 rpm. Cells (500 µL) were then harvested by centrifugation at 16,600× *g* for 10 min, and collected cells were assessed for staphyloxanthin production.

### 4.7. Sheep Red Blood Cell Hemolysis Assay

Sheep red blood cell hemolysis efficacies were analyzed using whole cultures of *S. aureus*, as described previously [48,49]. Briefly, *S. aureus* cells were diluted 1:100 in LB broth (1.5 × 10^7^ CFU/mL) with overnight culture, then incubated with or without CNMAs at 10, 20, or 50 µg/mL for 24 h with shaking at 250 rpm. Fresh whole sheep blood was separated by centrifugation at 3000× *g* for 5 min, and sheep red blood cells (MBcell, Seoul, Korea) were washed five times with sterile PBS and diluted in PBS (330 µL of red blood cells in 10 mL of PBS). S. aureus cultures (300 µL) were added to 1 mL of the diluted red blood cells. To measure hemolytic activities, mixtures of red blood and S. aureus were incubated at 250 rpm for 1 h at 37 °C. Absorbances of supernatants obtained by centrifugation at 10,000× *g* for 10 min were measured at 543 nm.

### 4.8. The Mixed UPEC/S. aureus Biofilm Model

To produce mixed UPEC/S. aureus biofilms, UPEC (5 × 10^6^ CFU/mL), and *S. aureus* (1.5 × 10^7^ CFU/mL) were inoculated into an NB/LB/water mix (1:1:1) in 96-well polystyrene plates (300 µL /well) and incubated for 24 h at 37 °C. Biofilm formation was then analyzed as described above.

### 4.9. Statistical Analysis

Results are presented as averages and standard deviations, and the significances of differences between averages were defined using one-way ANOVA followed by Dunnett’s test using SPSS version 23 (SPSS Inc., Chicago, IL, USA). Statistical significances were accepted for *p*-values < 0.05. All experiments were performed using at least two independent cultures.

## Figures and Tables

**Table 1:** The MICs of CNMAs against UPEC and *S. aureus*.

Test Compound	Structure	MIC (µg/mL) against UPEC	MIC (µg/mL) against *S. aureus*
*Trans*-CNMA	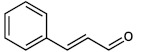	400	>400
4-BromoCNMA	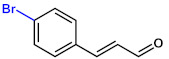	400	>400
4-ChloroCNMA	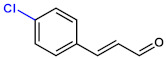	200	400
CNMA oxime	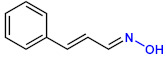	>400	>400
4-DimethylaminoCNMA	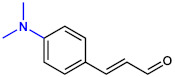	>400	>400
4-FluoroCNMA	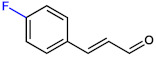	200	>400
2-MethoxyCNMA	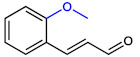	>400	>400
4-MethoxyCNMA	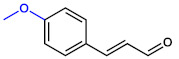	>400	>400
*α*-MethylCNMA	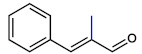	>400	>400
2-NitroCNMA	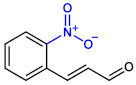	>400	>400
4-NitroCNMA	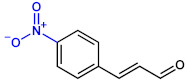	100	100

## Data Availability

All relevant data are within the manuscript.

## Supplementary Materials

The following supporting information can be downloaded at: https://www.mdpi.com/article/10.3390/ijms23137225/s1.
