# Supplementary material for: Antibiofilm Activities of Cinnamaldehyde Analogs against Uropathogenic Escherichia coli and Staphylococcus aureus
Source: Int J Mol Sci. 2022 Jun 29;23(13):7225. doi: 10.3390/ijms23137225 (PMC9267110; doi:10.3390/ijms23137225)
Supplement: Supplementary file 1 [file ijms-23-07225-s001.zip › ijms-1786771-supplementary.pdf]

# Antibiofilm Activities of Cinnamaldehyde Analogs against Uropathogenic *Escherichia coli* and *Staphylococcus aureus*

**Yeseul Kim [1,†], Sanghun Kim [1,†], Kiu-Hyung Cho [2], Jin-Hyung Lee [1,*] and Jintae Lee [1,*]**

[1] School of Chemical Engineering, Yeungnam University, Gyeongsan 38541, Korea; yeseul@ynu.ac.kr (Y.K.); minimo017@ynu.ac.kr (S.K.)

[2] Gyeongbuk Institute for Bioindustry, Andong 36618, Korea; khcho68@gmail.com

* Correspondence: jinhlee@ynu.ac.kr (J.-H.L.); jtlee@ynu.ac.kr (J.L.); Tel.: +82-53-810-3812 (J.-H.L.); +82-53-810-2533 (J.L.)

† These authors contributed equally to this work.

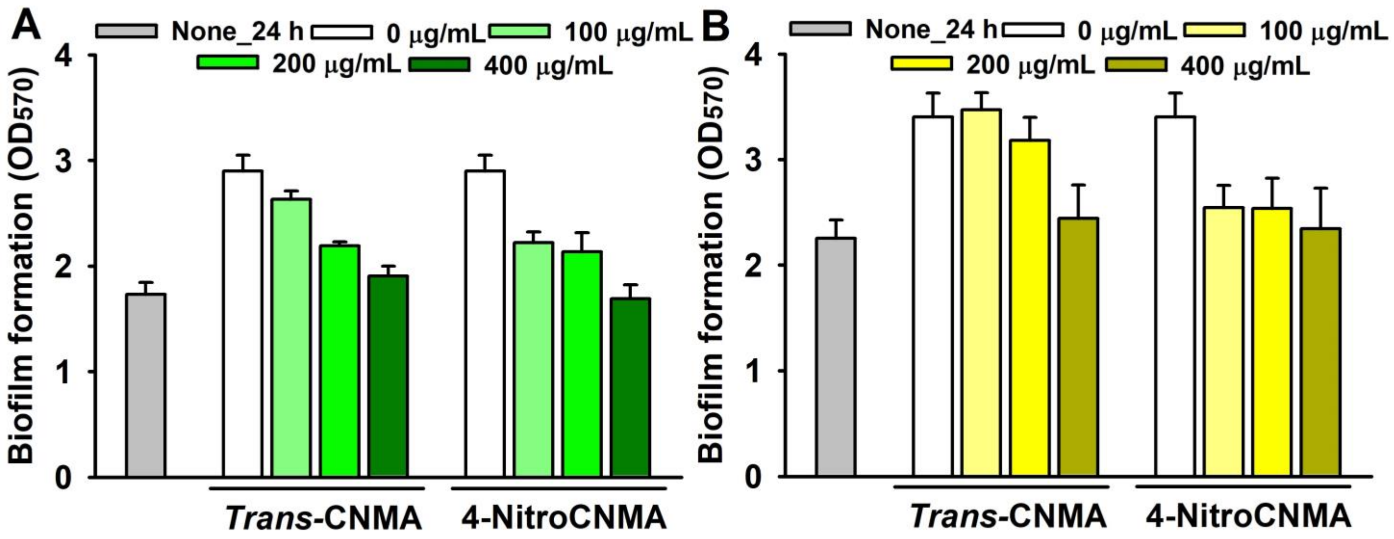


**Supplementary Figure S1.** Effects of *trans*-CNMA and 4-nitroCNMA on biofilm dispersal. Preformed biofilms of UPEC grown in NB medium (**A**) and of *S. aureus* grown in LB medium (**B**) for 24 h (grey bar) were treated with *trans*-CNMA or 4-nitroCNMA for 24 h. Biofilm formation was measured by crystal violet staining.

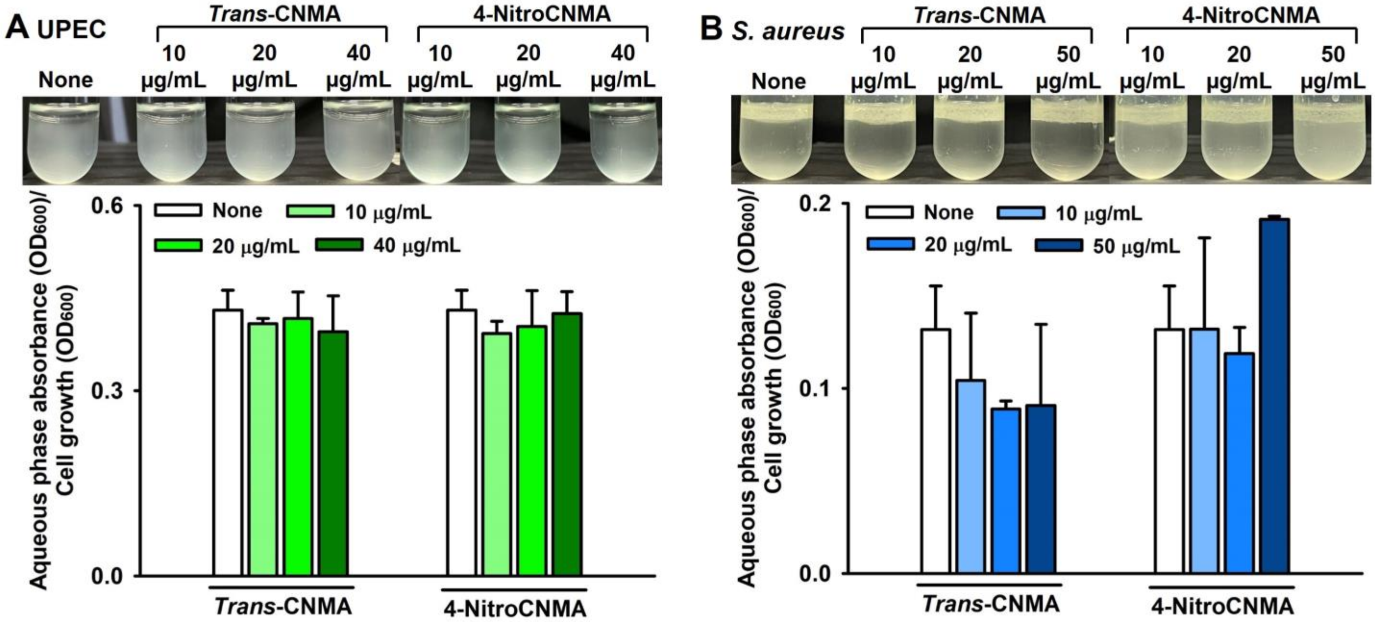


**Supplementary Figure S2.** Effects of *trans*-CNMA and 4-nitroCNMA on the hydrophobicity of UPEC (**A**) and *S. aureus* (**B**). Cell surface hydrophobicity of UPEC and *S. aureus* after treatment with or without *trans*-CNMA or 4-nitroCNMA at 10, 20 or 40 μg/mL (*S. aureus* 10, 20 or 50 μg/mL).

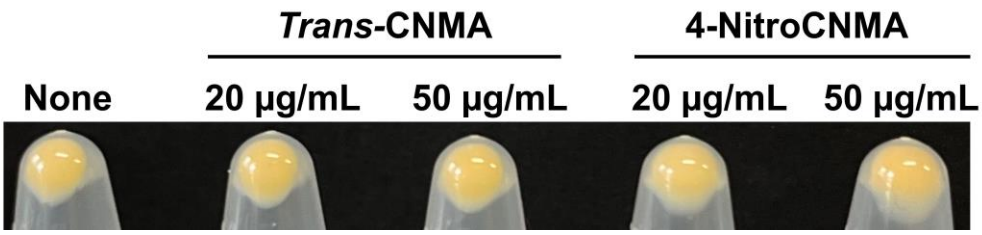


**Supplementary Figure S3.** Effects of *trans*-CNMA and 4-nitroCNMA on the production of staphyloxanthin in *S. aureus*.
